# CCL5-deficiency enhances intratumoral infiltration of CD8^+^ T cells in colorectal cancer

**DOI:** 10.1038/s41419-018-0796-2

**Published:** 2018-07-10

**Authors:** Shengbo Zhang, Ming Zhong, Chao Wang, Yanjie Xu, Wei-Qiang Gao, Yan Zhang

**Affiliations:** 1 State Key Laboratory of Oncogenes and Related Genes, Renji-Med-X Stem Cell Research Center, Renji Hospital, School of Biomedical Engineering, Shanghai Jiao Tong University, 200030 Shanghai, China; 2 Med-X Research Institute & School of Biomedical Engineering, Shanghai Jiao Tong University, 200030 Shanghai, China; 3 Department of Gastrointestinal Surgery, Renji Hospital, School of Medicine, Shanghai Jiao Tong University, 200127 Shanghai, China

## Abstract

Colorectal cancer (CRC) is the third most common solid tumor in the world and shows resistance to several immunotherapies, particularly immune checkpoint blockade which has therapeutic effects on many other types of cancer. Cytotoxic CD8^+^ T cell has been considered as one of the main populations of effector immune cells in antitumor immunity; however, the absence of CD8^+^ T cells in the central tumor area has become a major obstacle for solid tumor immunotherapy, particularly for CRC. Thus, novel therapeutic strategies that could promote CD8^+^ T cells to accumulate in the central tumor area are urgently needed. Here, we demonstrated that CCL5-deficiency delayed tumor growth and metastasis via facilitating CD8^+^ T cells to accumulate into tumor site in CRC mouse models. Furthermore, CCL5-deficiency could upregulate PD-1 and PD-L1 expression and reduce the resistance to anti-PD-1 antibody therapy in CRC mouse model. Mechanically, the results of RNA-sequencing, in vitro coculture system and hypoxia measurements demonstrated that knockdown of CCL5 could result in the metabolic disorders in CD11b^hi^F4/80^low^ TAMs and suppress the expression of S100a9 to promote the migration of CD8^+^ T cells in the tumor microenvironment. These findings were verified by the data of clinical samples from CRC patients, suggesting that CCL5 may provide a potential therapeutic target for the combined PD-1-immunotherapy of CRC.

## Introduction

Colorectal cancer (CRC) is the third most common cancer and the estimated number of new CRC cases was 71,420 in men and 64,010 in women in the USA in 2017^1,2^. The development of immunotherapies, including immune checkpoint inhibitors, chimeric antigen receptor (CAR)-expressing T cells and tumor vaccines, have made great progress in cancer treatment generally via liberating the killing power of T cells^3,4^. Cancer immunotherapies have shown considerable clinical benefits in various cancers; however, their effect on CRC are limited^5^. The non-T-cell-inflamed tumor, lack of T cells at the tumor microenvironment despite the presence of abundant active T cells circulating in the host, has been demonstrated to be a major immunotherapeutic barrier for CRC patients^6^. The presence of activated CD8^+^ T cells in tumor sites has been proved to be a significant positive prognostic marker for clinical response to immune checkpoints inhibitors in CRC^7–11^. Importantly, clinical response to anti-PD-1 Ab was found to occur almost exclusively in patients with pre-existing T cells infiltration^5,12,13^. Therefore, new methods to enhance intratumoral infiltration of CD8^+^ T cells are an urgent need for CRC patients to benefit from the immunotherapies.

In cancer, tumor-associated macrophages (TAMs) often contribute to cancer cell growth, invasiveness, and suppressing antitumor immunity^14^. More importantly, several studies have showed that macrophage are present in large number at the tumor sites, no matter if T cells are inflamed^15–17^. Our previous study had shown that CC chemokine ligand 5 (CCL5) could modulate the differentiation of myeloid-derived suppressor cells (MDSC) to promote tumor progression in luminal and triple-negative breast cancer^18^. In this study, we demonstrated that CCL5-deficiency inhibited tumor growth and metastasis of CRC by increasing the infiltration of CD8^+^ T cells into central tumor area. Mechanically, the reduced expression of S100a9 (S100 calcium-binding protein A9) in CD11b^hi^F4/80^low^ TAMs induced by CCL5-deficiency could contribute to this phenotype.

## Results

### CCL5-dificiciency inhibits the tumor progression in colorectal tumor models

**Fig. 1:**
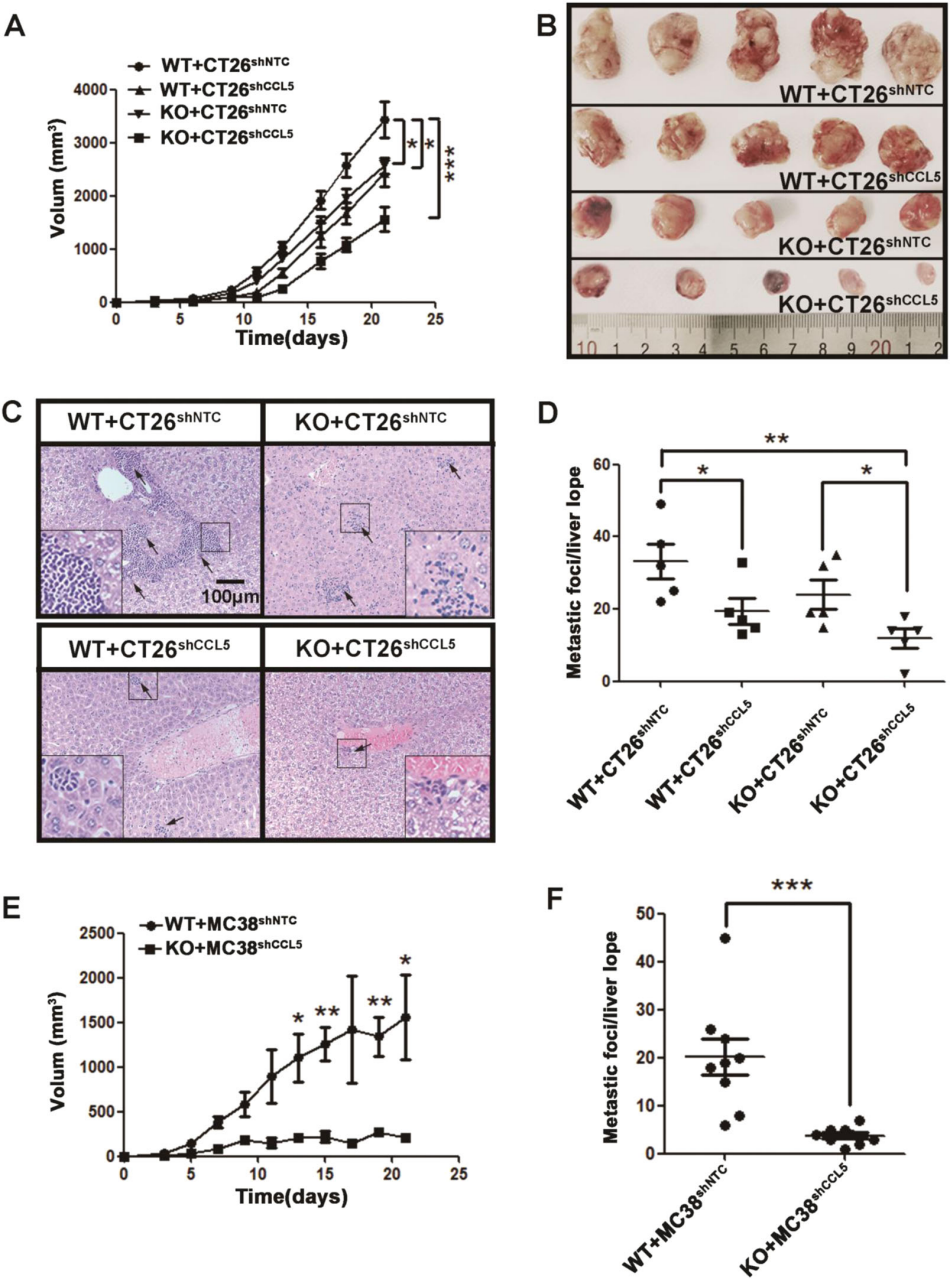
CCL5 promotes tumor growth and metastasis in mouse model of CRC. **a** Tumor growth curves of WT or KO BALB/c mice subcutaneously injected with CT26^shNTC^ or CT26^shCCL5^ tumor cells. Tumor growth was monitored every 2–3 days. *n* = 5 mice per group. **b** Representative pictures of tumors isolated from tumor-bearing mice after 3 weeks of cancer cell inoculation. **c** Histologic identification of liver metastasis of CT26 tumor cells by HE staining. **d** Quantitation of Fig. 1c by counting metastatic foci in every liver lobe. Each group contained five mice. **e** Tumor growth of WT and KO C57/B6 mice which were subcutaneous injected with MC38^shNTC^ or MC38^shCCL5^ tumor cells. Tumor growth was monitored every 2 days. **f** Quantitation of liver metastasis by counting metastatic foci in every liver lobe. Each group contained three mice and each mouse counted three hepatic lobules. *P < 0.05, **P < 0.01, ***P < 0.001. Data are represented as mean ± SEM

To explore the role of CCL5 on progress of CRC, CCL5 knockout (KO) and wild-type (WT) mice in BALB/c background were subcutaneously inoculated with CT26 colorectal carcinoma cells in which CCL5 expression was stably silenced via lentiviral small interfering RNA (WT + CT26^shCCL5^, KO + CT26^shCCL5^) or with control cell line (WT + CT26^shNTC^, KO + CT26^shNTC^). The efficiency of CCL5 knockdown was confirmed by RT-PCR (Supplementary Fig. 1A), Elisa (Supplementary Fig. 1B), and western blot (Supplementary Fig. 1C) in vitro, and by IHC in vivo (Supplementary Fig. 1D). Tumor volume was measured every 2 or 3 days until day 21. The results of growth curves showed that either knockout of host-derived or knockdown of tumor cell-derived CCL5 alone significantly decreased the tumor growth and deficiency of both host-derived and tumor cell-derived CCL5 dramatically inhibited the tumor growth, compared to the control group (Fig. 1a, b), even though the in vitro growth pattern of CT26^shCCL5^ was similar to that of CT26^shNTC^ (Supplementary Fig. 1E). For hepatic metastasis, the similar tendency was observed on the tumor burden in the liver and the number of metastasis foci (Fig. 1c, d). Based on the data that both host-derived CCL5 and tumor cell-derived CCL5 play important role on tumor progression in CRC, we chose KO + CT26^shCCL5^ (CCL5−/−) mice and the control group WT + CT26^shNTC^ (CCL5+/+) to explore the role of CCL5 in CRC in the following studies.

To further confirm the effect of CCL5 on CRC, we utilized orthotopic CT26 tumor model to verify our results. CT26^shNTC^ or CT26^shCCL5^ tumor cells were orthotopically injected into WT or KO mice with BALB/c background and was imaged on days 3, 6, and 10 via high-frequency ultrasound. The results showed that CCL5-deficiency could delay both tumor growth and metastasis of CT26 in orthotopic CRC model (Supplementary Fig. 2A, C and D). These phenotypes in CT26-BALB/c mice model were confirmed by subcutaneous and orthotopic transplanted MC38-C57/B6 mice model with CCL5-deficiency (Fig. 1e, f and Supplementary Fig. 1F, G and 2B, C and D). Collectively, CCL5-deficiency inhibits tumor growth and metastasis of CRC in mouse models.

### CCL5-deficiency promotes CD8^+^ T cells to accumulate in primary colorectal tumor site and enhances antitumor response of CD8^+^ T cells

**Fig. 2:**
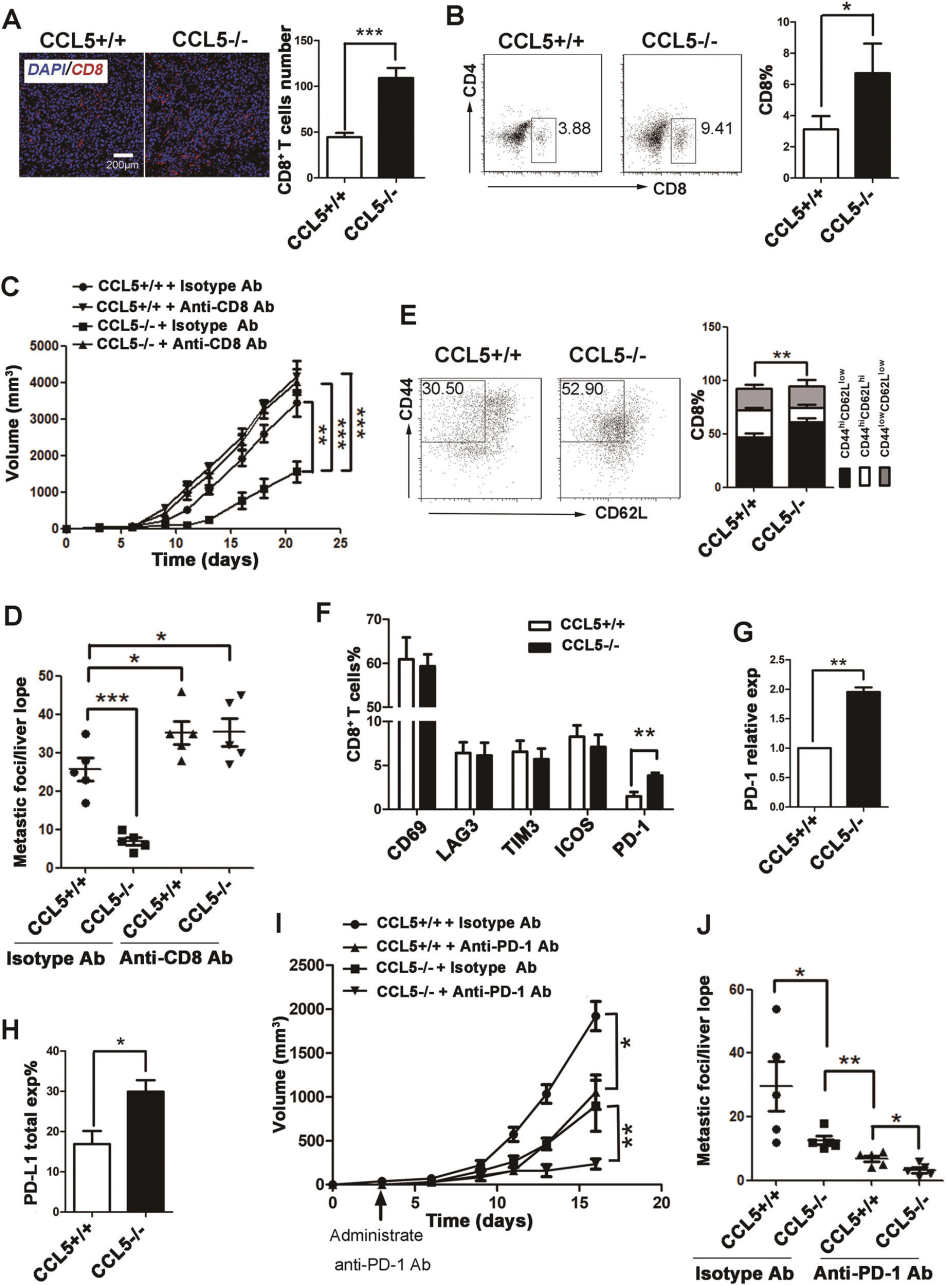
The phenotype of CD8^+^ T cells and decreased resistance to anti-PD-1 Ab in CCL5−/− mice. **a** Representative histological staining (left) and analysis (right) of CD8 in frozen section from the tumor of CCL5+/+ or CCL5−/− mice. **b** Representative flow cytometry plot (left) and analysis (right) of CD8^+^ T cells percentage in TILs of control group CCL5+/+ or CCL5−/− mice. *n* = 5 mice per group. **c** The tumor growth curves of CT26^shNTC^ and CT26^shCCL5^ tumor cells were injected into the back of female WT or KO mice treated with CD8 T-cell-depleting antibody. Tumor growth was monitored every 2–3 days. **d** Quantitation of liver metastasis determined by counting of foci per liver lobe. Each group contained five mice. **e** Representative flow cytometry plot (left) and analysis (right) of effector CD8^+^ T cells percentage among CD8^+^ cells in TILs of CCL5+/+ or CCL5−/− mice. Bar chart shows the FACS quantification of effector (CD8^+^CD44^hi^CD62L^low^), memory (CD8^+^CD44^hi^CD62L^hi^), and naïve (CD8^+^CD44^low^CD62L^low^) T-cell subpopulations. **f** FACS quantification of the percentage of CD69^+^, LAG3^+^, TIM3^+^, ICOS^+^, PD-1^+^ cells among CD8^+^ T cells in TILs of CCL5+/+ or CCL5−/− mice. **g** RT-PCR quantification of relative PD-1 mRNA expression in CD8^+^ T cells in TILs. **h** FACS quantification of the percentage of PD-L1^+^ cells in TILs. **i** The tumor growth curves of CT26^shNTC^ and CT26^shCCL5^ tumor cells were injected into the back of female WT or KO mice. Mice received anti-PD-1 monoclonal antibody i.p. on days 3, 6, 9, 12 post tumor injection. Tumor growth was monitored every 2–3 days. **j** Quantitation of liver metastasis determined by counting of foci per liver lobe after tumor burden of 40 days. Each group contained five mice. *P < 0.05, **P < 0.05, ***P < 0.001. Data are represented as mean ± SEM; *n* = 5 mice per group

The number of intratumoral infiltration of CD8^+^ T cells can be considered as a positive prognostic indicator in many human cancer types^19^. To characterize the role of CCL5 on CD8^+^ T cells tumor infiltration, tumor tissues from CCL5+/+ or CCL5−/− mice after 3 weeks of tumor challenge were examined by immunofluorescence and the number of tumor-infiltrating lymphocytes (TILs) were examined by FACS. The results of immunofluorescence staining showed that the number of CD8^+^ T cells infiltrated in the tumor of CCL5−/− mice was significantly higher than that in the control group (Fig. 2a), which was confirmed by the data of flow cytometry (FC) in CT26 mouse model (Fig. 2b). However, there were no significant differences in the percentage of other lymphocytes, such as CD4^+^ T cells (Supplementary Fig. 3A) and B cells (Supplementary Fig. 3B) between CCL5+/+ and CCL5−/− groups. To figure out the reasons on the accumulation of CD8^+^ T cells in the tumor site in CCL5−/− mice, we first detected the percentage of CD8^+^ T cells in circulating blood in CCL5+/+ and CCL5−/− mice after 3 weeks of tumor challenge. The results revealed that the number of CD8^+^ T cells was decreased in the blood of CCL5−/− group compared with that of CCL5+/+ group (Supplementary Fig. 3C), suggesting that there was an enhanced migration of CD8^+^ T cells into the tumor sites in CCL5−/− mice. These phenotypes were also verified by another CRC mouse model (MC38-C57B6) (Supplementary Fig. 3D). These results demonstrated that CCL5 contributes to tumor progress in CRC models possibly via inhibiting the accumulation of CD8^+^ T cells in tumor sites.

To demonstrate the important role of CD8^+^ T cells in CCL5-deficiency-mediated tumor regression of CRC, we performed antibody-mediated CD8^+^ T-cell depletion in CCL5+/+ and CCL5−/− mice to examine the tumor growth and metastasis. The efficiency of CD8^+^ T-cell depletion was determined by FACS analysis of peripheral blood (Supplementary Fig. 3E). Depletion of CD8^+^ T cells dramatically enhances the tumor growth and liver metastasis in CCL5−/− mice, compared to the isotype Ab treatment group, and their antitumor activity of CCL5-deficiency in CRC was lost (Fig. 2c, d). These results implied that CD8^+^ T cells mediated the inhibitory effect of CCL5-deficiency on tumor progression of CRC.

The CD8^+^ T-cell response to tumor-specific antigen can typically be divided into three phases: priming and expansion, resolution and contraction, and memory^20^. During these phases, naïve CD8^+^ T cells (CD44^low^CD62L^hi^) proliferate and differentiate into effector CD8^+^ T cells (CD44^hi^CD62L^low^) and memory CD8^+^ T cells (CD44^hi^CD62L^hi^)^21^. To figure out which subset of CD8^+^ T cells was affected by CCL5-deficiency, we analyzed the composition of subpopulation of CD8^+^ T cells infiltrating into tumor area by FC. The results showed that the proportion of effector CD8^+^ T cells, but not that of memory CD8^+^ T cells, was significantly increased in CCL5−/− mice compared to that of CCL5+/+ mice (Fig. 2e). In addition, we also examined the expression of activate marker CD69 on tumor-infiltrating CD8^+^ T cells and the results showed that the expression level of CD69 on CD8^+^ T cells has no significant difference between CCL5+/+ and CCL5−/− mice (Fig. 2f and Supplementary Fig. 4A). Collectively, these results demonstrated that CCL5-deficienty promotes the migration of CD8^+^ T cells into tumor and increases the antitumor immune response of CD8^+^ T cells to inhibit tumor growth and metastasis.

### CCL5-deficiency decreases the resistance to PD-1-immunotherapy in mouse colorectal tumor model

The immune checkpoint molecules include inhibitory checkpoint molecules such as PD-1, lymphocyte-activation gene 3 (LAG-3), T-cell immunoglobulin mucin-3 (TIM-3), and stimulatory checkpoint molecules such as the inducible costimulatory molecule (ICOS) that is always expressed on the surface of activated T cells^22^. Immune checkpoint inhibitor therapy has shown important clinical advances due to its function on releasing the activities of T cells^23^. To examine the effect of CCL5-deficiency on the expression level of these immune checkpoint molecules, tumor-infiltrated CD8^+^ T cells were analyzed by FC. The results showed that there was no significant differential expression of LAG-3, TIM-3, and ICOS on CD8^+^ T cells between CCL5+/+ and CCL5−/− mice either from tumor or spleen (Fig. 2f and Supplementary Fig. 4B-D). In contrast, the expression level of PD-1 on the surface of CD8^+^ T cells was significantly enhanced in the CCL5−/− mice compared with that of CCL5+/+ mice (Fig. 2f and Supplementary Fig. 4E), which was confirmed by the data of RT-PCR (Fig. 2g).

Programmed death receptor ligand 1 (PD-L1), the known ligand for the PD-1 receptor, is a member of the immune checkpoint family B7, and is expressed on the surface of antigen-presenting cells and tumor cells^24^. So we tested the expression level of PD-L1 on the surface of single cells which were collected from tumor tissue by FC after 3 weeks of tumor challenge. Figure 2h and Supplementary Fig. 4F showed that the expression levels of PD-L1 was higher in CCL5−/− mice than that of CCL5+/+ mice, revealing that CCL5-deficiency upregulates the expression of both PD-1 and PD-L1 in tumor tissue.

The anti-PD-1 monoclonal antibody has shown notable objective responses after administration in many advanced tumors^5^. However, there are still about 67% CRC patients showing resistance to anti-PD-1 Ab treatment^25^. To determine the effect of CCL5-deficiency on anti-PD-1 Ab resistance in CRC, we intraperitoneally injected 200 μg anti-PD-1 Ab into CCL5+/+ and CCL5−/− mice every 3–4 days from day 3 to day 16 after tumor challenge. Data showed that both tumor progress (Fig. 2i and Supplementary Fig 5) and hepatic metastasis of colorectal tumor (Fig. 2j) were dramatically decreased in CCL5−/− mice treated with anti-PD-1 Ab compared with anti-PD-1-Ab-treated CCL5+/+ mice. In conclusion, these results showed that CCL5-deficiency could reduce the resistance to anti-PD-1 antibody therapy in CRC.

### CCL5-deficiency changes the phenotype of CD11b^hi^F4/80^low^ TAMs

**Fig. 3:**
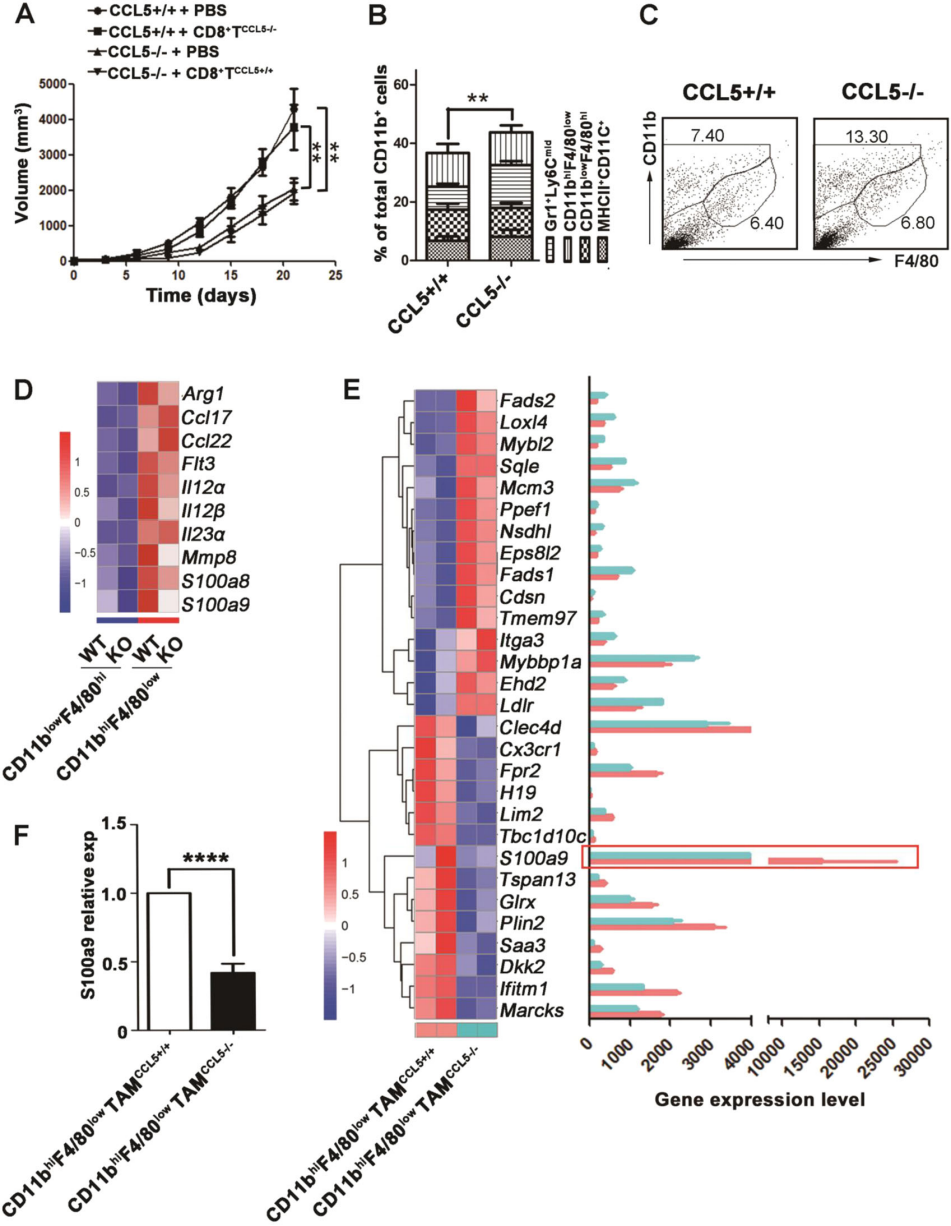
The phenotype of CD11b^hi^F4/80^low^ TAMs is changed in CCL5−/− mice. **a** At 7 and 12 days following the initial tumor challenge, adoptive transfer of CD8^+^ T cells (6×10^6^) was done. Tumor growth was measured 2–3 times a week. **b** Flow cytometry analysis of myeloid populations of TILs. *n* = 5 mice per group. **c** Flow cytometry analysis of TAMs in tumor from CCL5+/+ or CCL5−/− mice. **d** Transcriptomic profiling and gene expression level of CD11b^low^F4/80^hi^ TAMs and CD11b^hi^F4/80^low^ TAMs by RNA-seq, which were separated from the tumor of CCL5+/+ and CCL5−/− mice. **e** Transcriptomic profiling and gene expression level of CD11b^hi^F4/80^low^ TAMs^CCL5+/+^ and CD11b^hi^F4/80^low^ TAMs^CCL5−/−^ by RNA-seq. **f** RT-PCR quantification of S100a9 gene relative expression in CD11b^hi^F4/80^low^ TAMs^CCL5+/+^ and CD11b^hi^F4/80^low^ TAMs^CCL5−/−^. **P < 0.01, ****P < 0.0001. Data are represented as mean ± SEM

Due to the chemo attractive activity of CCL5 on lymphocytes, we first tested the direct effect of CCL5 on CD8^+^ T cells migration by trans-well experiments in vitro. However, the results showed that CCL5 had no direct effects on the migration of CD8^+^ T cells (Supplementary Fig. 6). To explore the possibility of intrinsic CCL5-deficiency in CD8^+^ T cells to affect the migration and antitumor activity of CD8^+^ T cells, we adoptively transferred CCL5+/+ CD8^+^ T cells or CCL5−/− CD8^+^ T cells isolated from tumor-bearing mice into CCL5−/− mice or CCL5+/+ mice, respectively. The FACS data demonstrated that adoptive transfer of CD8^+^ T cells had no effect on the number of intratumoral CD8^+^ T cells in both CCL5−/− and CCL5+/+ mice, suggesting that intrinsic CCL5 protein in CD8^+^ T cells does not play an important role in the migration of these cells (Supplementary Fig. 4). As expected, adoptive transfer of CCL5+/+ CD8^+^ T cells did not enhance tumor growth and metastasis of CCL5−/− mice and vice versa that CCL5−/− CD8^+^ T cell did not change the phenotype of CCL5+/+ mice (Fig. 3a and Supplementary Fig. 8).

According to our previous study, the absence of CCL5 causes the accumulation of abnormal Ly6C^hi^Ly6G^+^ MDSC that loses its immunosuppressive activity in triple-negative 4T1 mammary carcinoma model^18^. To figure out if myeloid cells are involved in CCL5-deficiency-induced intratumoral infiltration of CD8^+^ T cells, the major subsets of tumor-infiltrated myeloid cells, granulocytes (CD11b^+^Gr1^+^Ly6C^mid^), CD11b^hi^F4/80^low^ TAMs, CD11b^low^F4/80^hi^ TAMs and DCs (MHCII^+^CD11b^+^), were examined by FC. The results showed that the proportions of granulocytes, CD11b^low^F4/80^hi^ TAMs, and DCs had no significant difference between CCL5+/+ and CCL5−/− mice, except for that of CD11b^hi^F4/80^low^ TAMs (Fig. 3b, c). To further characterize the phenotype of TAMs, the CD11b^hi^F4/80^low^ TAMs and CD11b^low^F4/80^hi^ TAMs were isolated from tumor tissues of CCL5+/+ and CCL5−/− mice by FACS. Figure 3d demonstrated that CD11b^hi^F4/80^low^ TAMs have higher expression levels of cytokines than CD11b^low^F4/80^hi^ TAMs, regardless of CCL5+/+ or CCL5−/− mice. Recent studies showed that CD11b^hi^F4/80^low^ TAMs are monocyte-derived macrophages and play important role in tumor progress^26,27^. Next, we focus on the function of CD11b^hi^F4/80^low^ TAMs isolated from tumor sites of CCL5+/+ mice and CCL5−/− mice and their global transcriptional profiles were analyzed using RNA-sequencing (Supplementary Fig. 9A). Figure 3e showed that CCL5-deficiency affected the metabolism-related genes (*Fads1, Fads2, Lox14, Sqle, Nsdh1, GLrx, Marcks*), the proliferation-related genes (*Mybl2, Mcm3, Ppef1, Ldlr, Plin2*) and the cell motility-related genes (*Cdsn, Itga3, H19, Limd2*). These results of RNA-sequencing were verified by RT-PCR (Supplementary Fig. 9B-E). Importantly, the result of Fig. 3e showed that the expression level of S100a9 was significantly decreased in CD11b^hi^F4/80^low^ TAM^CCL5−/−^ (CD11b^hi^F4/80^low^ cells isolated from CCL5−/− mice) compared to CD11b^hi^F4/80^low^ TAM^CCL5+/+^ (CD11b^hi^F4/80^low^ cells isolated from CCL5+/+ mice), which was confirmed by RT-PCR (Fig. 3f). In conclusion, CCL5-deficiency dramatically changed the phenotype of CD11b^hi^F4/80^low^ TAMs, and the expression level of S100a9 was dramatically decreased in CD11b^hi^F4/80^low^TAM^CCL5−/−^ compared to the control group.

### CD11b^hi^F4/80^low^ TAMs from CCL5-deficiency mice promote the migration of CD8^+^ T cells via inhibition of S100a9 secretion

**Fig. 4:**
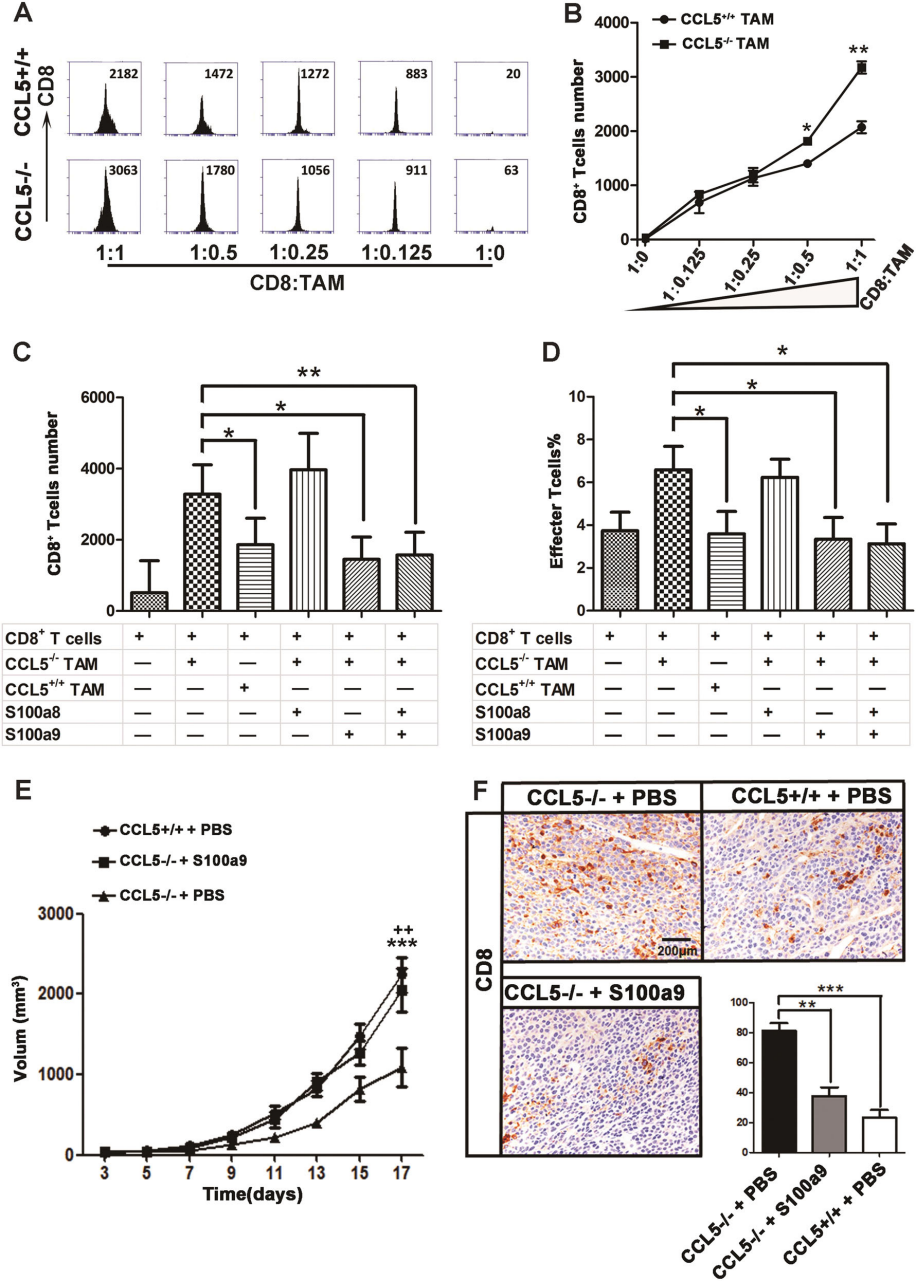
CD11b^hi^F4/80^low^ TAMs^CCL5+/+^ inhibit infiltration of CD8^+^ T cells via secreting S100a9. **a** CD8^+^ T cells isolated from spleen of BALB/c mice and CD11b^hi^F4/80^low^ TAMs separated from TILs of CCL5+/+ or CCL5−/− mice by FACS. They were cocultured in 48-well trans-well plates for 24 h and CD8^+^ T cells were counted by FACS. **b** Quantification of (**a)**. **c** S00a8 and/or S100a9 (200 ng/mL) were added in the trans-well cocultured system; after 24 h, CD8^+^ T cells were counted by FACS. **d** S00a8 and/or S100a9 (200 ng/mL) were added in the cocultured system; after 24 h, FACS quantification of effector CD8^+^ T cells (CD44^hi^ and CD62L^low^). **e** Tumor growth in CCL5+/+ and CCL5−/− mice injected with S100a9. Mice received S100a9 (100ug/mL) i.p. every 2–3 days from day 3 to day 15. Tumor growth was monitored every 2 days. *n* = 3 mice per group. **f** Representative immunohistochemistry staining and analysis of CD8 in tumor section from CCL5+/+ or CCL5−/− mice which were injected with S100a9 or PBS. *n* = 3 mice per group. *P < 0.05, **P or ^++^ <0.01, ***P *<* 0.001; **e** *CCL5+/++ PBS versus CCL5−/− + PBS and ^+^CCL5+/++ S100a9 versus CCL5−/− + PBS. Data are represented as mean ± SEM; results are representative of three independent experiments

S100a9, a calcium-binding protein, is considered as proinflammatory mediator to suppress the migration of CD8^+^ T cells^28–30^. It has been demonstrated that the expression of S100a9 in MDSC is essential for the development of colorectal tumors^31^. To figure out if the decreased level of S100a9 was involved in the activity of CD11b^hi^F4/80^low^ TAM^CCL5−/−^ on the intratumoral accumulation of CD8^+^ T cell, we first established the transwell coculture system. Results showed that CD11b^hi^F4/80^low^ TAM^CCL5−/−^ could significantly promote CD8^+^ T-cell migration at a ratio of 1:0.5 and 1:1 in vitro (Fig. 4a, b). Next, we examined the role of S100a9 in the promigrative ability of CD11b^hi^F4/80^low^ TAM^CCL5−/−^ on CD8^+^ T cells. Since S100a9 and its binding partner S100a8 are members of the S100 calcium-binding family of proteins which form a heterocomplex, termed S100a8/a9 or calprotectin^32^, we also examined the effects of S100a8 on CD8^+^ T-cell migration. The results showed that the promigrative potential of CD11b^hi^F4/80^low^ TAM^CCL5−/−^ on CD8^+^ T cells can be counteracted by adding extra S100a9 alone or combine with S100a8 in the trans-well coculture system, while S100a8 alone had no effect on it (Fig. 4c).

To further explore if CD11b^hi^F4/80^low^ TAMs^CCL5−/−^ could affect the formation of effector CD8^+^ T cells, the subpopulation of CD8^+^ T cells was analyzed by FC after 24 h coculture. Figure 4d showed that S100a9 alone or combined with S100a8 can inhibit the generation of effector CD8^+^ T cells induced by CD11b^hi^F4/80^low^ TAMs^CCL5−/−^, while S100a9 had no effect on the apoptosis of CD8^+^ T cells (Supplementary Fig. 10). To further confirm the effect of S100a9 on the CCL5-induced CRC progress, S100a9 protein was intraperitoneally injected into CCL5−/− mice every 3 days for four times. The tumor growth rate and hepatic metastasis of CCL5−/− mice were increased after S100a9 treatment compared to CCL5−/− mice treated with PBS (Fig. 4e and Supplementary Fig. 11). At the same time, the results of IHC showed that the number of intratumoral CD8^+^ T cells was decreased in CCL5−/− mice injected with S100a9 protein, compared to those injected with PBS (Fig. 4f). The results demonstrated that decreased level of S100a9 in CD11b^hi^F4/80^low^ TAMs contributes to the CCL5-deficiency-induced intratumoral migration of CD8^+^ T cells.

### The infiltration of CD8^+^ T cells into central hypoxic area of tumor is enhanced by CD11b^hi^F4/80^low^ TAMsCCL5^−^^/^^−^ in vivo

**Fig. 5:**
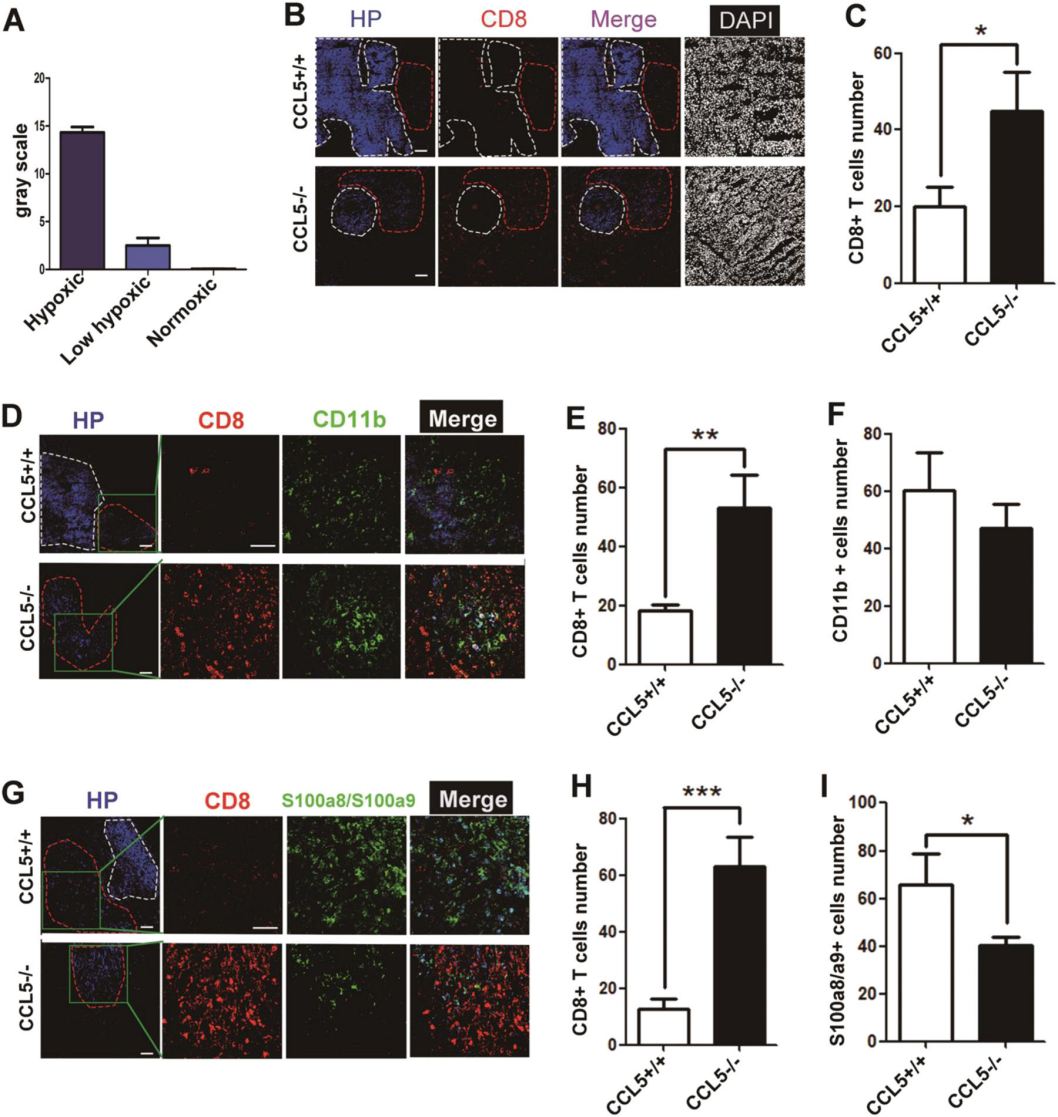
The accumulation of CD8^+^ T cells in central area of tumor site was enhanced by CD11b^+^F4/80^low^ TAMs in CCL5-deficient mice. **a** ImageJ analysis fluorescence intensity of HP-1 to determine hypoxic (white line), low hypoxic (red line), and normoxic area at 30 μs exposure time. **b** The location of CD8^+^ T cell in the tumor site examined by immunofluorescence staining. 3-week tumor-bearing mice were injected with HP-1. Tumor sections were stained with anti-HP-1, CD8, and 4′,6-diamidino-2-phenylindole (DAPI). **c** Analysis of CD8^+^ T cells number in the tumor’s low hypoxic area of CCL5+/+ or CCL5−/− mice. *n* = 5 mice per group. **d** The location of CD8^+^ T cell and TAMs in the tumor site examined by immunofluorescence staining. Tumor sections were stained for HP-1, CD8, CD11b, and DAPI. **e** Quantitating the number of CD8^+^ T cells in (**d**). **f** Quantitating the number of CD11b^+^ cells in (**d**). **g** The location of CD8^+^ T cell and S100a8/a9 in the tumor site examined by immunofluorescence staining. Tumor sections were stained for HP-1, CD8, S100a8/S100a9, and DAPI. **h** Quantitating the number of CD8^+^ T cells in (**g**). **i** Quantitating the number of S100a8/a9^+^ cells in (**g**). Data are represented as mean ± SEM; *n* = 5 mice per group

To visualize the location of CD11b^hi^F4/80^low^ TAMs and CD8^+^ T cells in the tumor site, we used immunofluorescence technique to detect the location of those cells in the tumor tissues of CCL5+/+ mice and CCL5−/− mice. To visualize the hypoxic area in tumor, Pimonidazole (HP-1), which is reductively active in an oxygen-dependent manner and covalently binds to thiol-containing proteins in hypoxic cells^33^, was used. Based on fluorescence intensity of HP-1 at the same exposure time, tumor section was separated into normoxic area, low hypoxic area, and hypoxic area (Fig. 5a). Figure 5b shows that tumor indeed contained a large number of hypoxic areas, especially in CCL5+/+ mice. The results also demonstrated that although CD8^+^ T cells could not infiltrate into the tumor hypoxic area in either CCL5+/+ or CCL5−/− mice, there were a lot of CD8^+^ T cells infiltrating into low hypoxic areas in the tumor of CCL5−/− mice but not in those of CCL5+/+ mice (Fig. 5b, c).

To further verify the role of CD11b^hi^F4/80^low^ TAMs^CCL5−/−^ on the accumulation of CD8^+^ T cells in vivo, tumor sections were stained for CD11b, CD8, and HP-1. Data showed that many CD11b^hi^ cells, which were also F4/80^low^ (Supplementary Fig. 12), mainly aggregated in the low hypoxic area in both CCL5+/+ and CCL5−/− mice (Fig. 5d−f). What’s important, there were more S100a8/a9 cells in the low hypoxic area in the tumor of CCL5+/+ mice than CCL5−/− mice (Fig. 5g−i). In conclusion, CD11b^hi^F4/80^low^ TAMs suppress CD8^+^ T-cell infiltration into tumor low hypoxic area of tumor tissue via secretion of S100a8/a9.

### CRC patients with higher expression level of CCL5 have lower number of CD8^+^ T cells in tumor sites

**Fig. 6:**
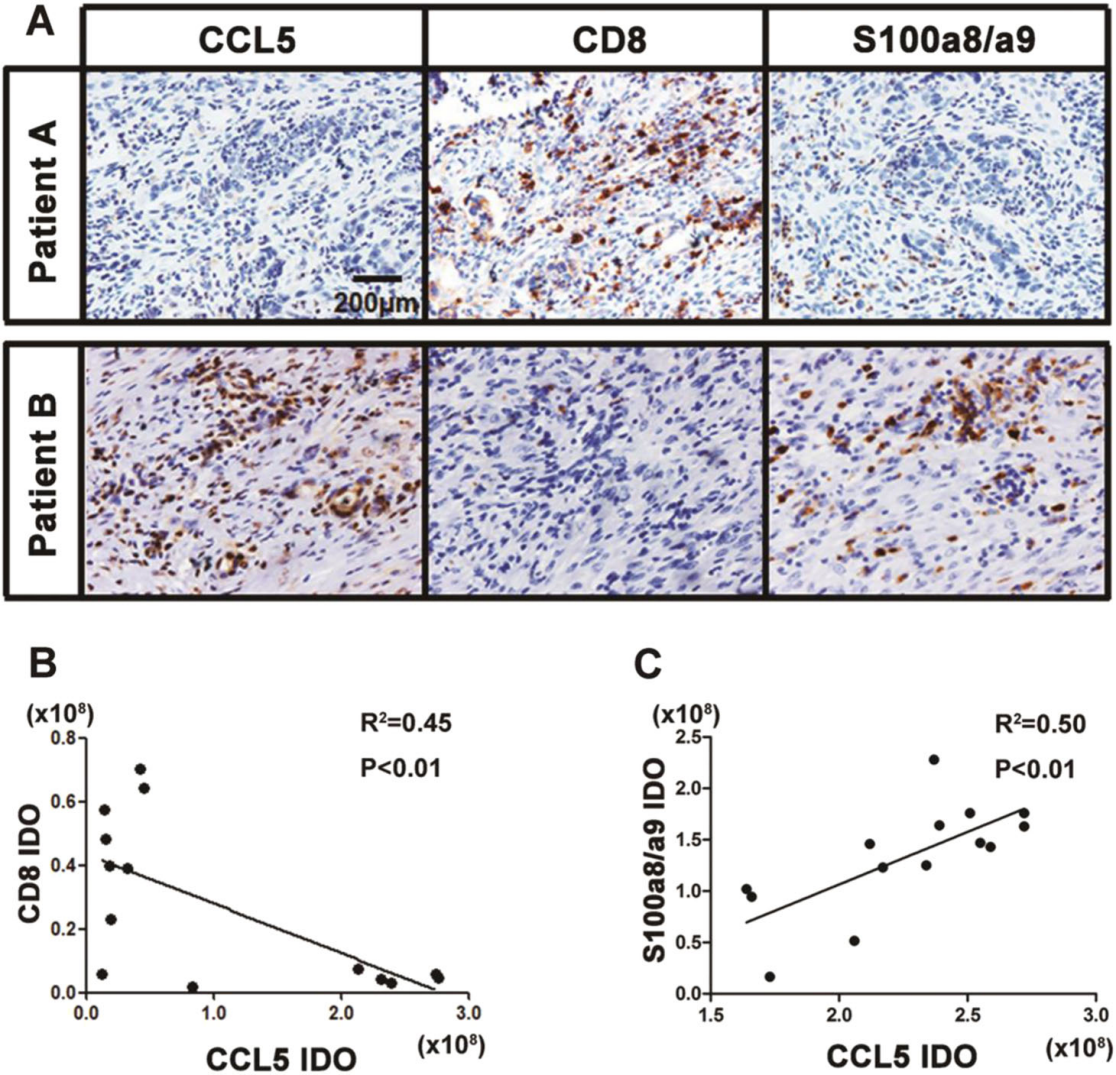
CRC patients with higher CCL5 have lower number of CD8^+^ T cells infiltrating into tumor sites. **a** Histologic identification of human CCL5, CD8, and S100a8/a9 in the continuous paraffin section of CRC tumor. **b** Correlation between CCL5 and CD8 in 14 CRC patients. Expression of CCL5 and S100a8/a9 were identified by IHC. Quantitative analysis of histological staining using ImageJ. **c** Correlation between CCL5 and S100a8/a9 in 15 CRC patients. Expression of CCL5 and S100a8/a9 were identified by IHC. Quantitative analysis of histological staining using ImageJ. Data were analyzed using Pearson correlation analysis

To provide further evidence on the conclusion that CCL5 suppresses CD8^+^ T cells accumulation via secreting S100a8/a9, we examined the correlation between the expression level of CCL5 and S100a8/a9 and the number of CD8^+^ T cells infiltrated in the tumor of CRC patients. We found that the expression of CCL5 and S100a9 were significantly high in CRC patients who has low number of CD8^+^ T cells infiltrating into tumor tissue (Fig. 6a), and the correlation analysis revealed that the expression level of CCL5 was negatively correlated with the number of intratumoral CD8^+^ T cells (Fig. 6b), but was positively correlated with S100a9 expression (Fig. 6c). We also performed analysis on the mRNA expression level of CCL5 and S100a9 with the data gotten from the public TCGA dataset (*n* = 83) and further verified the positive correlation between these two proteins in CRC (Supplementary Fig. 13A). We also demonstrated that the expression level of S100a9 was significantly increased in the tumor site compared to the normal colon of CRC patients (normal = 51, CRC = 383; Supplementary Fig. 13B). These data suggested that CCL5 might also play an important role in suppressing CD8^+^ T cells to infiltrate into tumor site by S100a9 in CRC patients.

## Discussion

The amount of CD8^+^ T cells present in the tumor area has been considered as a positive signal in the treatment of CRC patients^9,10,34,35^. Here, we reported that CCL5-deficiency dramatically inhibited S100a9 secretion in CD11b^hi^F4/80^low^ TAMs, which contributed to the ability of CD11b^hi^F4/80^low^ TAMs^CCL5−/−^ in promoting the infiltration of CD8^+^ T cells into the central tumor area.

CCL5 is a secreted small molecular protein that can dissociate into blood and tumor microenvironment^36^, and performs its function via binding to its receptors (CCR1, CCR3, and CCR5) on target cell surfaces^36^. Previous studies have shown that the expression level of CCL5 is associated with tumor growth and metastasis formation in several types of cancers^37–42^. And it can be expressed by macrophages, T cells, tubular epithelium, synovial fibroblasts, and certain types of cancer cells^43,44^. Our and another researcher’s previous work have demonstrated that either tumor-derived or host-derived CCL5 could play an important role in tumor progression^18,39,45–47^. In this study, we chose to knockdown both tumor-derived and host-derived CCL5 to decrease the amount of CCL5 as much as possible in mouse models.

Tumor-associated myeloid cells have been reported to enhance invasive and metastatic capabilities of malignant cells through production of cytokines, such as EGF and TGFβ^48^. Our previous study also demonstrated that CCL5 could induce the formation of MDSC to promote tumor growth and metastasis in triple-negative breast cancer. The present work provides evidence that CCL5 can act through CD11b^hi^F4/80^low^ TAMs indirectly, rather than directly through the CD8^+^ T cells, for inhibiting the accumulation of CD8^+^ T cells into the central area of CRC.

Hypoxia has been shown to be a major factor in impeding the migration of CD8^+^ T cells into central tumor area^49^. Our results demonstrated that CD11b^hi^F4/80^low^ TAMs mechanism located in the low hypoxic area could inhibit the intratumoral infiltration of CD8^+^ T cells by secreting S100a9 protein. S100a9 is expressed predominantly by myeloid cells and dramatic upregulation of this protein has been observed in many tumors, including breast, ovarian, bladder, gastric, colorectal, pancreatic, thyroid, and skin cancers^50–52^. Grebhardt et al. identified hypoxia and HIF-1 as novel regulators of S100a9 expression in prostate cancer^29^. We firstly showed that CCL5-deficiency in the tumor microenvironment could significantly downregulate the expression level of S100a9 in CD11b^hi^S100a9^hi^ TAMs in CRC. For mechanisms on this phenotype, we hypothesized that the inhibition on the expression of *Saa3* in CD11b^hi^S100a9^hi^ TAMs^CCL5−/−^ by CCL5-deficiency might contribute to the decreased expression level of S100a9, which will need to be investigated in future studies.

Considering a rationale using a combination strategy to induce the accumulation of CD8^+^ T cells in tumor sites and to reactivate CD8^+^ T cell on which PD-1/PD-L1 acts might allow us to develop a more effective anticancer therapy. And application of CCL5-deficiency by CCL5-neutralizing antibody in combination with anti-PD-1 antibody might extend the survival of patients with CRC.

## Materials and methods

### Patients

Primary tumor samples of 23 CRC patients were obtained from Renji Hospital of Shanghai Jiao Tong University (Shanghai, China).

### Mice, cell line

WT BALB/c and C57/B6 mice were purchased from the Jackson Laboratories (Bar Harbor, ME, USA). CCL5-KO BALB/c and CCL5-KO C57/B6 mice were acquired as previously described^18^. KO mice have no morphologically or functionally overt abnormal phenotype. Genotyping was done using PCR of tail DNAs. BALB/c CT26 and C57/B6 MC38 colorectal carcinoma cell lines were purchased from ATCC. 1×10^6^ cells were subcutaneously injected into the back of female BALB/c or C57/B6 mouse at 6–8 weeks of age. Tumors were collected when they reached the size of 1.5−2.0 cm in diameter.

### Study approval

All animal studies were reviewed and approved by the Institutional Animal Care and Use Committee of Shanghai Jiao Tong University. All human samples were collected with the informed consent of the patients and the procedures were approved by Renji Hospital of Shanghai Jiao Tong University (Renji (2017) N017).

### Cell sorting

For tumor-infiltrating leukocytes (TILs), tumors were digested into single cells with collagenase type II (0.5 mg/mL), collagenase type IV (0.5 mg/mL), hyaluronidase (10 U/mL), and DNase I (0.01 mg/mL) (Worthington, USA) for 2 h at 37 °C. The dissociated cells were collected, lysed by RBC lysis buffer. For TAMs, the cells were incubated with 7-AAD, F4/80, and CD11b monoclonal antibodies. For CD8^+^ T cells, spleens were mechanically dissociated and strained though a 40-μm nylon mesh to produce a single-cell suspension. The cells were incubated with CD8a monoclonal antibody. The positive cells were next sorted by FC on BD FACSAria using BD FACSDiva software. The purity of the isolated subpopulations regularly exceeded 90%. All antibodies for cell sorting and FC were purchased from BD Biosciences or eBiosciences (Supplemental Table 1).

### Flow cytometry

The concentration of a single-cell suspension which was obtained from blood, spleen, lymph, or tumor was adjusted to 1×10^6^–10^7^/mL. Single-cell suspensions were stained for surface marker. Samples were tested by BD Accuri C6.

### Administration of anti-PD-1 in vivo

The in vivo effective neutralizing antibody against murine PD-1 mAb was purchased from BioXcell (USA). An irrelevant isotype-matched IgG was used as the control. CRC CCL5+/+ or CCL5−/− mice were treated via intraperitoneal injection (i.p.) with 200 µg of either anti-PD-1 or control IgG suspended in 500 µl of sterile PBS on days 3, 6, 9, 12 post tumor injection.

### Hypoxia measurements

For hypoxia staining, mice were injected with 80 mg/kg body weight pimonidazole (hypoxyprobe-1 (HP-1), HP, Inc.). Two hours later, tumors were snap-frozen, sections were acetone fixed, and stained by HPI-100Kit (HPI, USA). Pictures were acquired with Confocal (Carl Zeiss) and ZEN 2012 software (Carl Zeiss). ImageJ was used to semiquantitatively analyze normoxic, hypoxic, and low hypoxic areas.

### Quantitative RT-PCR

Cell lines or tumor tissues were homogenized with 1 mL TRI reagent to extract total RNA. cDNA was synthesized by reverse transcription of total RNA (Epicentre). The expression levels of target gene were determined using TaqMan Universal Master Mix II and GAPDH TaqMan probe was used as an internal control. The formula is 2^(−[(Ctgene−CtGAPDH)−(Ctgene−CtGAPDH)]).

### Immunofluorescence

Freezing tissues were cut into 4 μm sections, then fixed with 4% paraformaldehyde in PBS, permeabilized with 0.2% Triton X-100 in PBS, blocked in 10% donkey serum for 1 h at room temperature, followed by incubation with antibodies against CD11b (BD, USA), F4/80 (Abcam, USA), CD8 (R&D, USA), S100a8/S100a9 (Abcam), overnight at 4 °C, and detected with goat anti-rat Alexa 647(1:1000, eBioscience, USA), donkey anti-rabbit Alexa 546 (1:100, Sigma, USA), donkey anti-chicken Alexa 488-conjugated antibodies (1:500, Abcam). 4′,6-diamidino-2-phenylindole (DAPI, 1/100, Roche) was used to stain nuclei and images were acquired with Confocal (Carl Zeiss) and ZEN 2012 software (Carl Zeiss).

### Histology and immunohistochemistry

Formalin-fixed paraffin-embedded tissues were cut into 7 μm sections for efficiency testing. Specimens were hematoxylin and eosin stained according to standard protocols. Primary antibodies include CD8 (1:200, Abcam), CCL5 (human, 1:200, Abcam; mouse, 1:100, NOVUS, USA), S100a8/a9 (1:200, Abcam) and dilutions for IHC. For visualization, either horseradish peroxidase (HRP)-labeled secondary was used and detection was performed by following the manual of the Bond Polymer Refine Detection Kit on a Bond Max staining roboter (Leica). Quantitative analysis of histological staining was done using ImageJ^53^.

### Coculture of CD8^+^ T cells and CD11b^hi^F4/80^low^ TAMs

Coculture of CD8^+^ T cells and CD11b^hi^F4/80^low^ TAMs was performed using two different methods; a direct cell contact and transwell (contact-independent) system. CD8^+^ T cells were isolated from spleen of BALB/c mice by FACS. CD11b^hi^F4/80^low^ TAMs were isolated from the tumor of CCL5+/+ mice or CCL5−/− mice by FACS, which inoculated for 3 weeks. For the transwell experiments, 500 µL of media was placed on the CD11b^hi^F4/80^low^ TAMs, a 5 µM polyester membrane transwell (Corning, USA) was added to the well, and then 200 µL of media that contain 3×10^5^ CD8^+^ T cells were added on top of the transwell. S100a8 and/or S100a9 (200 ng/mL, Sino Biological, China) were added or not in the bottom of the transwell. Finally, the cells were cocultured for 24 h, then counted or tested by FACS. For the direct cell contact experiments, trans-well insert was removed from the 48-well plates.

### Lentivirus production and transfection

The pIKO.1 or pIKO.1-CCL5-shRNA1/2/3 plasmid was co-transfected into HEK-293T cells along with the packaging plasmid psPAX2 and the envelope plasmid pMD2G using Lipofectamine 2000 (Invitrogen, USA). Virus particles were harvested 48 h after cotransfection and were individually used to infect CT26 or MC38 cells. The cells were then harvested at 3 days after infection for western blotting and RT-PCR validation.

### Proliferation

Cell proliferation was assessed by a CCK-8 assay (Dojindo, Japan) according to the manufacturer’s instructions. Absorbance was measured using a microplate reader (HIDEX, Finland).

### Enzyme-linked immunosorbent assay (ELISA)

ELISA development reagents (DuoSet kit) for mouse CCL5 were purchased from R&D (USA), and the assay was performed according to the manufacturer’s instructions. Absorbance was measured using a microplate reader (HIDEX, Finland).

### Western blotting

Cells were lysed using the RIPA buffer (Sigma) along with Protease Inhibitor Cocktail (Calbiochem). The lysates were electrophoresed in a 10% SDS-PAGE gel and transferred to polyvinylidene fluoride membranes (Millipore). The membranes were incubated with primary antibody at 4 °C overnight after blocking using 5% milk. Then, the corresponding HRP-conjugated immunoglobulin G was incubated at room temperature for 1 h. Finally, signals were visualized with an enhanced chemi-luminescence kit (Millipore) and then analyzed using ImageJ software (NIT, Bethesda, Maryland). The CCL5 primary antibodies were purchased from CST (USA); β-actin (CST) was used as control.

### Methods for RNA-seq

Our raw sequence reads (GSE105042) were initially processed by FASTQC for quality control, then adapter sequences and poor-quality reads were removed by using CUTADAPT. Quality filtered reads were then mapped to mm9 using STAR^54^, and only uniquely mapped reads were kept. Read counts were calculated by using htseq-count^55^. Differential gene expression analysis was done using R package DESeq2^56^.

### Experimental procedures orthotopic CRC tumor model and ultrasound imaging

The experimental procedures orthotopic CRC tumor model was performed according to previous reports^57^. We monitored tumor growth by using a high-frequency ultrasound imaging system two times a week using Vevo 2100 ultrasound device (Fujifilm Visual Sonics, Toronto, Canada) equipped with a high-frequency (30 MHz) linear array transducer. This system was generously provided by Dr. Xianting Ding (Shanghai Jiao Tong University, China). Isoflurane was used to anesthetize mice and kept them on a heated platform. We then removed abdominal hair using depilatory cream and applied ultrasound gel on the skin. We identified tumor in the cecum as a low echoic mass and acquired images using the transducer.

### CD8^+^ T-cell depletion

CD8^+^ T-cell depletion was achieved following IP injection of 200 μg of CD8 depleting antibody (2.43. Bioxcell) into female age-matched BALB/c mice on days −1, 2, 7, 12. CT26^shNTC^ or CT26^shCCL5^ cells were injected into left flanks and tumor growth measured. The extent of T-cell depletion was determined at the end of the study using FACS (Supplementary Fig. 3E).

### Generation of CD8^+^ T cells and adoptive CD8^+^ T cells CRC model

Splenic CD8^+^ T cells were isolated from CCL5+/+ or CCL5−/− mice by negative selection (MACS, Miltenyi Biotec) and were activated (10^5^/mL) in medium containing RPMI 1640 with 10% fetal bovine serum, IL-2 (50 units/mL), anti-CD3^+^ and anti-CD28^+^ monoclonal antibody (30 ng/mL, R&D Systems) for 6 h. At days 7 or 12 after tumor injection, CCL5+/+ mice received an i.v. injection of CD8^+^ T^CCL5−/−^ cells (6×10^6^ cells per mouse). CCL5−/− mice received an i.v. injection of CD8^+^ T^CCL5+/+^ cells (6×10^6^ cells per mouse). Control groups of mice were injected with PBS.

### Statistical analysis

The Student’s *t* test was used to analyze the data. Results are given as mean ± SEM unless otherwise indicated. *P* values < 0.05 were considered significant.

## Electronic supplementary material

Supplementary data clean
