# Supplementary material for: CCL5-deficiency enhances intratumoral infiltration of CD8+ T cells in colorectal cancer
Source: Cell Death Dis. 2018 Jul 10;9(7):766. doi: 10.1038/s41419-018-0796-2 (PMC6039518; doi:10.1038/s41419-018-0796-2)
Supplement: Supplementary file 1 — Supplementary data clean [file 41419_2018_796_MOESM1_ESM.pdf]

Supplementary information

CCL5-deficiency enhances intratumoral infiltration of $CD8^+$ T cells in colorectal cancer

Shengbo Zhang [1,2,4], Ming Zhong [3,4], Chao Wang [1,2], Yanjie Xu [1,2], Wei-Qiang Gao [1,2]*, Yan Zhang [1,2]*

Supplemental Figures.

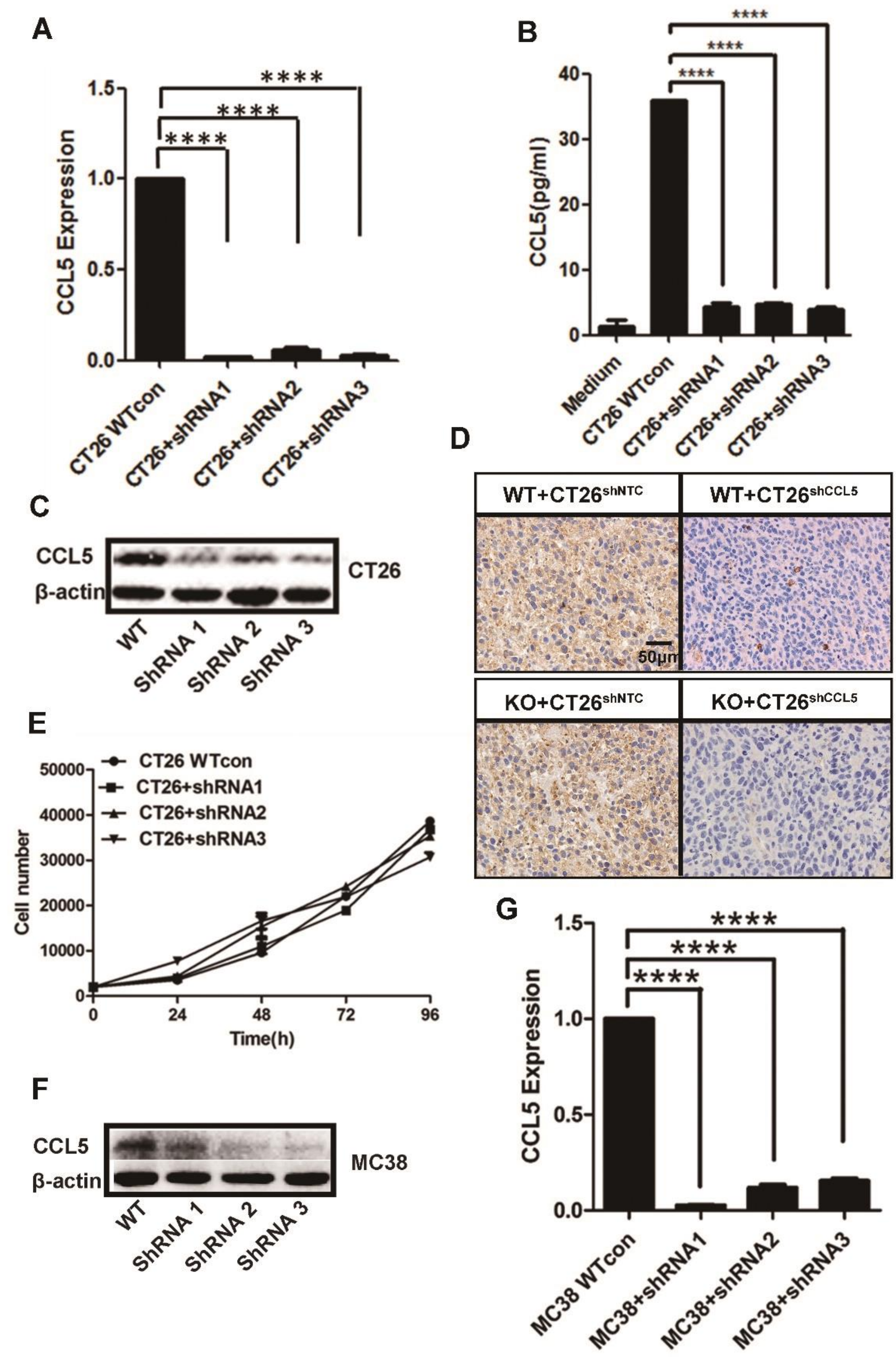

Supplementary figure 1. Confirmation of CCL5-knock down in CT26 and MC38 cells (A) CCL5 expression was quantified by RT-PCR at CT26^{shNTC} (CT26 WTcon) or CT26^{shCCL5} (CT26+shRNA 1, shRNA2 or shRNA3) cells. (B) CCL5 expression was quantified by Elisa at CT26^{shNTC} or CT26^{shCCL5} cells'

culture supernatant. (C) CCL5 expression was quantified by Western blot at $CT26^{shNTC}$ or $CT26^{shCCL5}$ cells. (D) Histologic identification of murine CCL5 staining in the tumor site by immunohistochemistry staining. (E) $CT26^{shNTC}$ and $CT26^{shCCL5}$ cells growth rate was measured by CCK8. (F) CCL5 expression was quantified by Western blot at $MC38^{shNTC}$ or $MC38^{shCCL5}$ cells. (G) CCL5 expression was quantified by RT-PCR at $MC38^{shNTC}$ or $MC38^{shCCL5}$ cells.
*****P*<0.0001. Data are represented as mean±SEM.

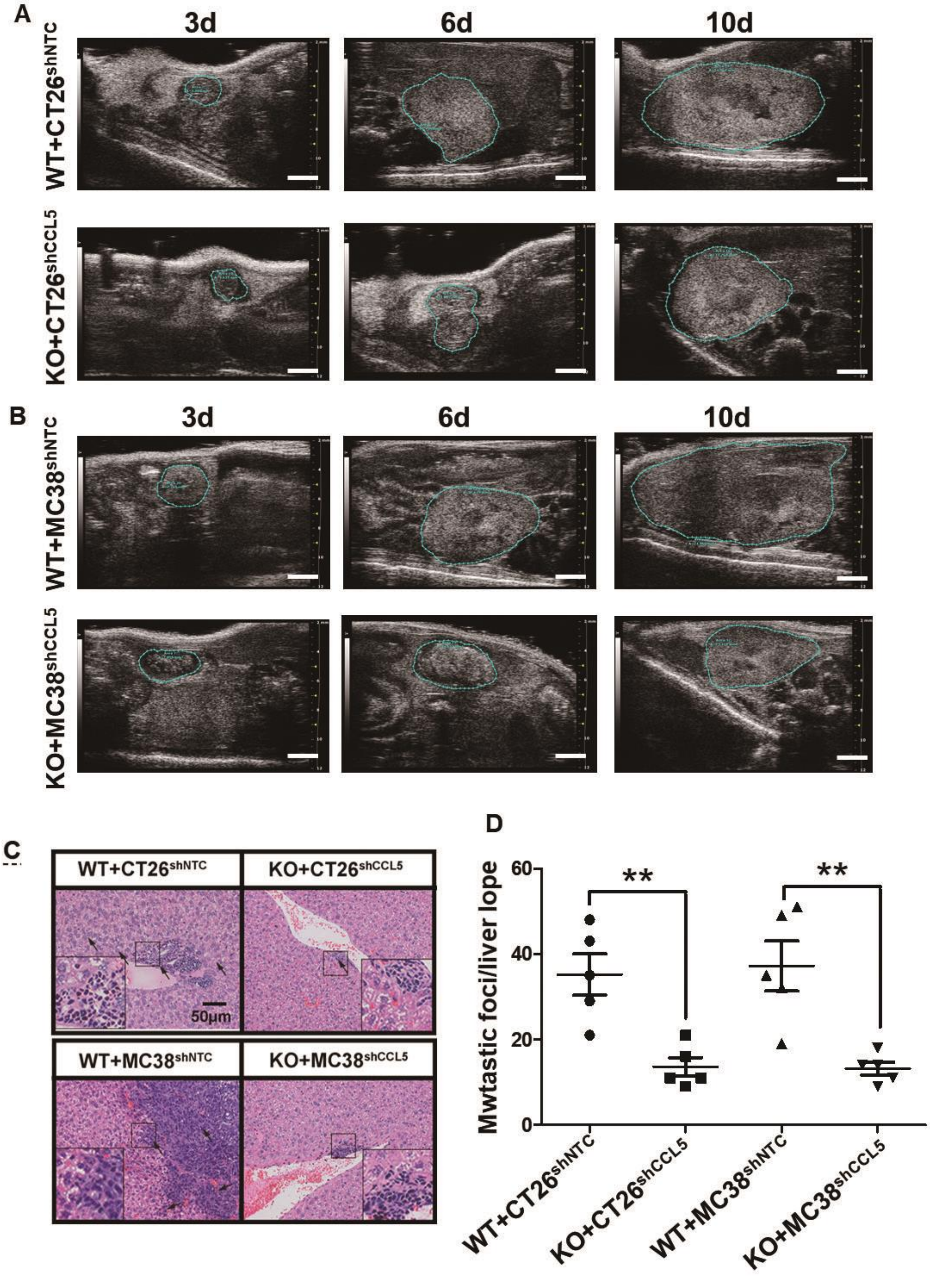

Supplementary figure 2. CCL5 promotes tumor growth and metastasis in orthotopic mouse model of CRC. (A) Representative ultrasound images of orthotopic CT26 tumors in BALB/c mice. WT or KO BALB/c mice were orthotopic injected with $CT26^{shNTC}$ or $CT26^{shCCL5}$ tumor cells and imaged on

days 3, 6, and 10 after injection. (Scale bar: 1 μm.) (B) Representative ultrasound images of orthotopic MC38 tumors in C57/B6 mice. WT or KO C57/B6 mice were orthotopically injected with $MC38^{shNTC}$ or $MC38^{shCCL5}$ tumor cells and imaged on days 3, 6, and 10 after injection. (Scale bar: 1 μm.) (C) Histologic identification of liver metastasis of orthotopic CT26 and MC38 CRC mouse model by HE staining. (D) Quantitation of liver metastasis determined by counting of foci per liver lobe. Each group contained five mice.

**$P<0.01$; Data are represented as mean±SEM.

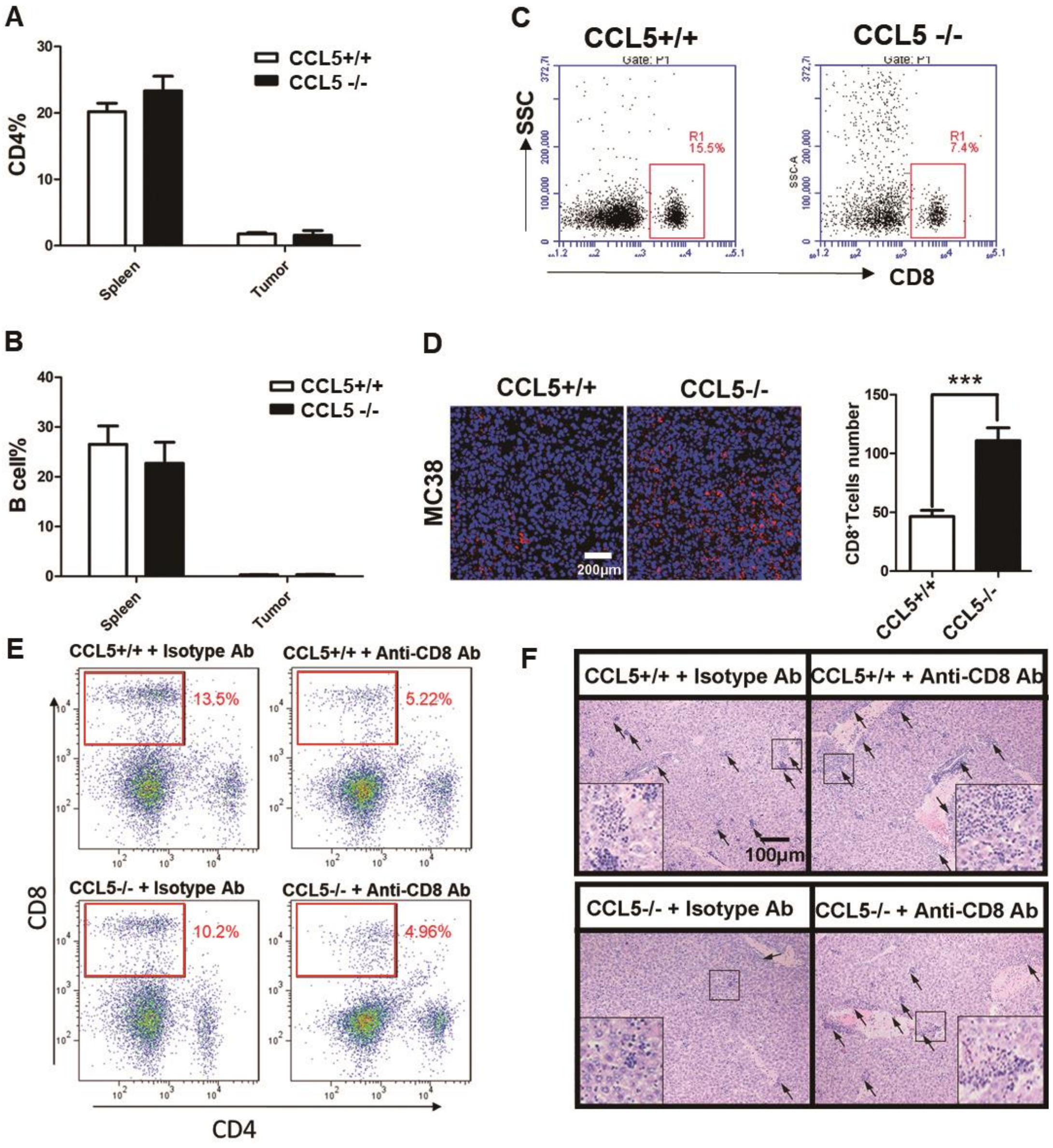

Supplementary figure 3. CD8⁺ T cells play a major role in CRC regression. (A) FACS quantification of CD4⁺ T cells in TILs and spleen. (B) FACS quantification of B cells in TILs and spleen, for CD20 marker. (C) The percentage of absolute number of CD8⁺ T cells in blood of CCL5+/+ and CCL5-/- mice and determined by flow cytometry. (D) Representative histological staining of CD8 (Red) and DAPI (Blue) in frozen section from the tumor of CCL5+/+ or CCL5-/- mice, n=5 mice per group. (E) Identification of CD8⁺ T cells depletion via Flow Cytometry. (F) Histologic identification of liver metastasis of CD8⁺ T cells depleted CRC mouse model by HE staining.

****P*<0.001. Data are represented as mean±SEM.

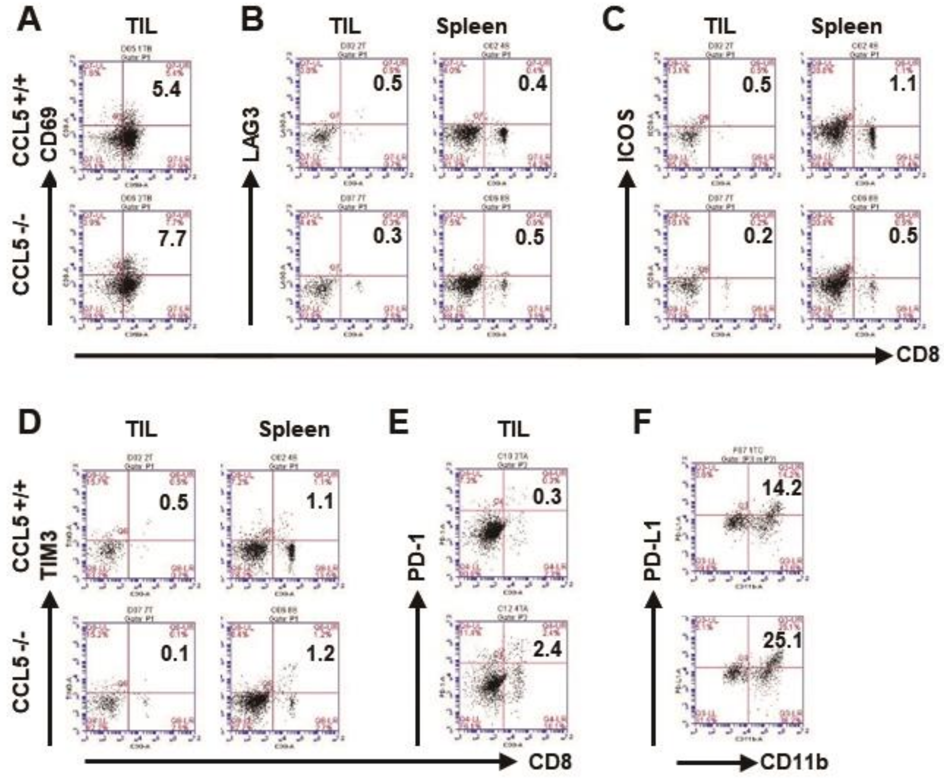

Supplementary figure 4. **The percentage of PD-1 or PD-L1 positive immune cells were increased after CCL5 knock out in CT26 tumor site.** (A) The percentage of absolute number of $CD69^{+}CD8^{+}$ T cells in TILs of CCL5+/+ or CCL5-/- mice and determined by flow cytometry. (B-D) The percentage of absolute number of $LAG3^{+}$/$ICOS^{+}$ or $TIM3^{+}$ and $CD8^{+}$ T cells in TILs and spleen of CCL5+/+ or CCL5-/- mice and determined by flow cytometry. (E) The percentage of absolute number of PD-$1^{+}CD8^{+}$ cells in TILs of CCL5+/+ or CCL5-/- mice and determined by flow cytometry. (F) The percentage of absolute number of PD-$L1^{+}CD11b^{+}$ cells in CCL5+/+ or CCL5-/- mice and determined by flow cytometry.

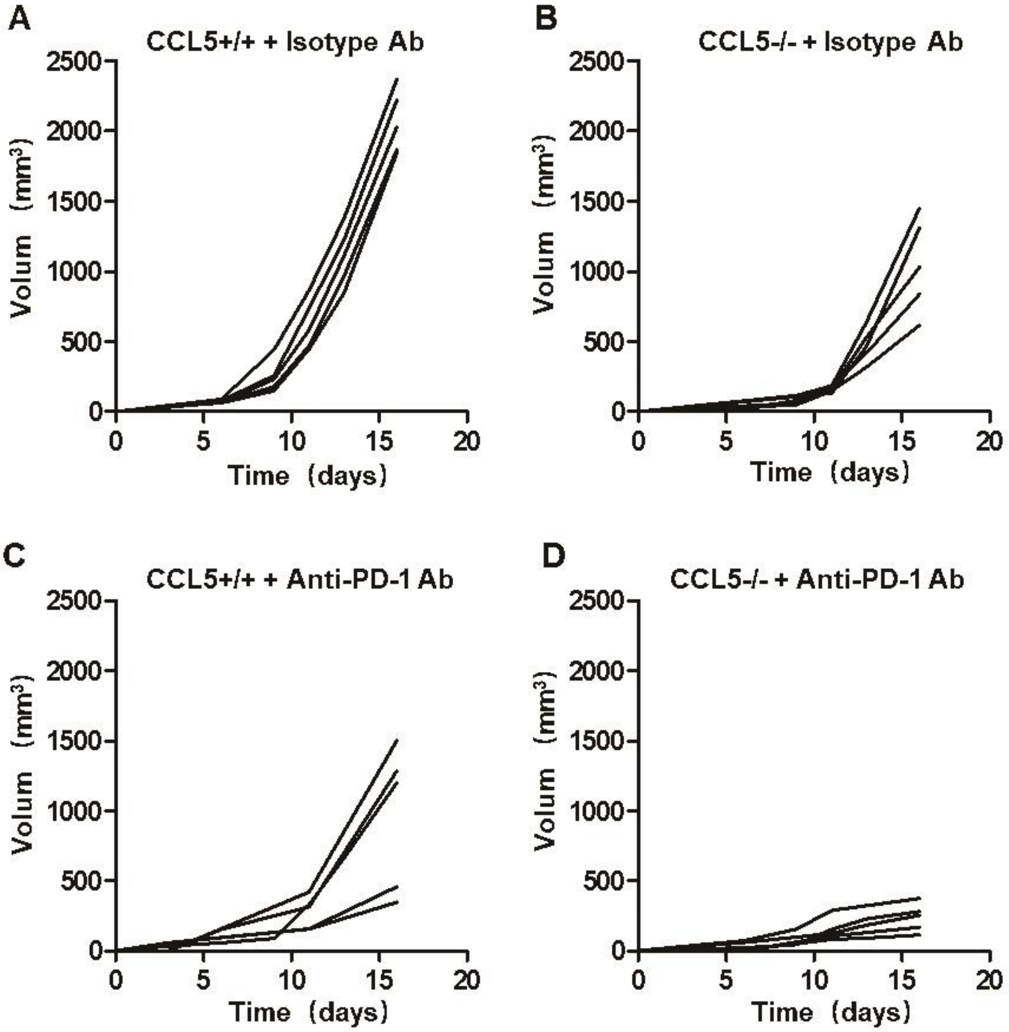

Supplementary figure 5. **Decreased resistance to anti-PD-1 Ab in CCL5-/- mice.** (A-D) Single growth curves of Fig 2 I. Each curve represents each mouse.

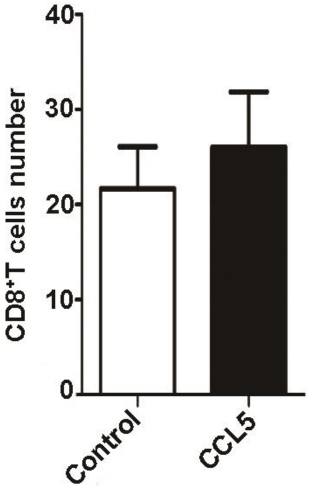

Supplementary figure 6. **The chemo attractive ability of CCL5 on CD8$^+$ T cells in vitro.** CD8$^+$ T cells were isolated from spleen of WT BALB/c mice by FACS, then co-cultured 24 hours in 24 well trans-well plates and 3x10^5 CD8$^+$ T cells/per trans-well chamber, the concentration of CCL5 is 10ng/ml. CD8$^+$ T cells were counted by FACS.

Data are represented as mean±SEM, results are representative of 3 independent experiments.

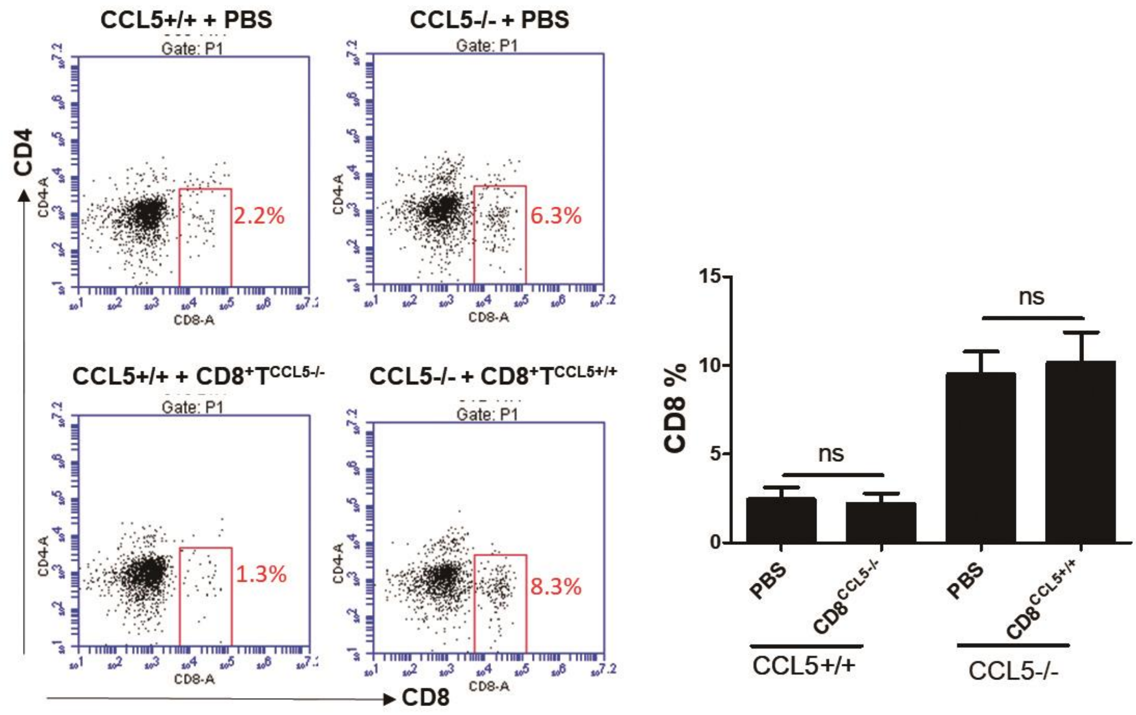

Supplementary figure 7. Intrinsic CCL5 deficiency in CD8$^+$ T cells doesn't affect the migration of CD8$^+$ T cells. At 7 and 12 days following the initial tumor challenge, adoptive transfer of CD8$^+$ T cells (6×10^6) was done. The mice were sacrificed in day 22. The data represents flow cytometry plot (left) and analysis (right) of CD8$^+$ T cells percentage in TILs of CCL5+/+ or CCL5-/- mice. n=5 mice per group.

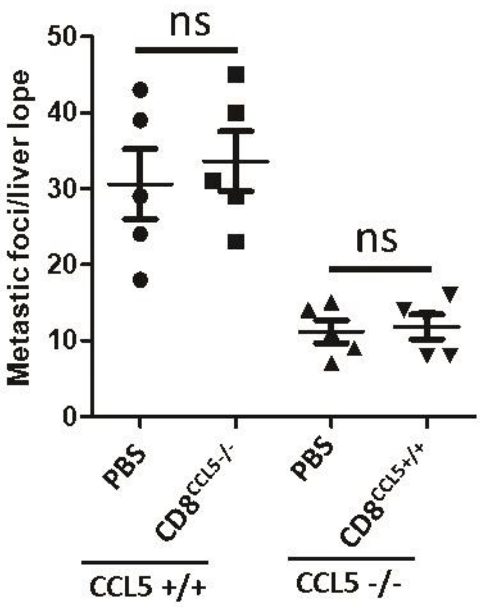

Supplementary figure 8. Intrinsic CCL5 deficiency in CD8⁺ T cells has no effect on the CRC liver metastasis. Quantitation of liver metastasis determined by counting of foci per liver lobe. Each group contained five mice.

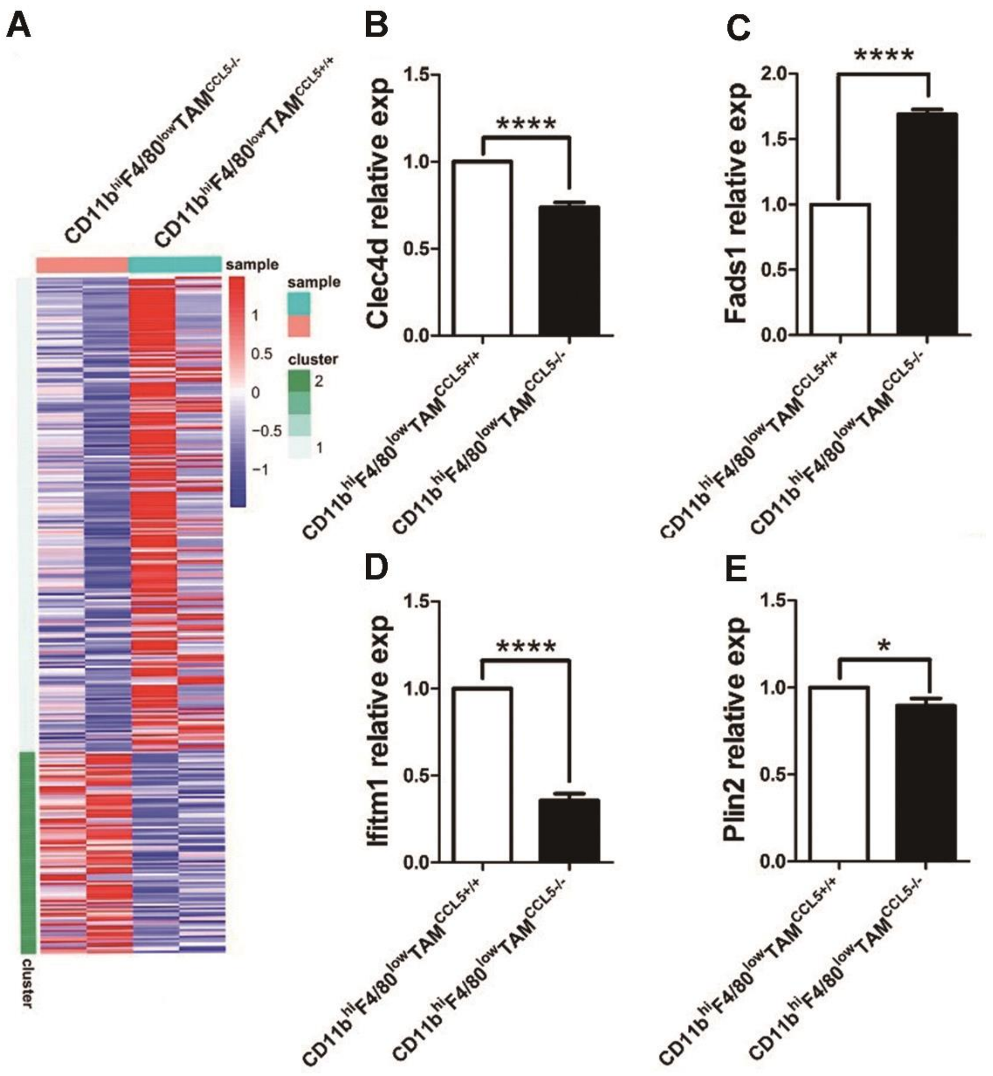

Supplementary figure 9. **Transcriptomic profiling of CD11b^{hi}F4/80^{low} TAMs$^{CCL5+/+}$ and CD11b^{hi}F4/80^{low} TAMs$^{CCL5-/-}$ cells.** A Heatmap of differential expression analysis of RNA sequencing data of CD11b^{hi}F4/80^{low} TAMs$^{CCL5+/+}$ and CD11b^{hi}F4/80^{low} TAMs$^{CCL5-/-}$ cells. B-E Q-PCR identify accuracy of the RNA-seq result by test the relative expression of Clec4d, Fads1, Plin2, Ifitm1.

**P*<0.05, ***P*<0.05, ****P*<0.001; Data are represented as mean±SEM; results are representative of 3 independent experiments.

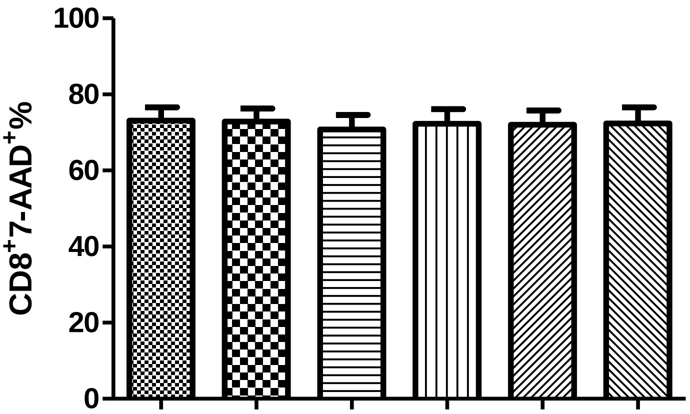

$CD8^+$ T cells	+	+	+	+	+	+
$CCL5^{-/-}$ TAM	—	+	—	+	+	+
$CCL5^{+/+}$ TAM	—	—	+	—	—	—
S100a8	—	—	—	+	—	+
S100a9	—	—	—	—	+	+

Supplementary figure 10. The apoptosis of $CD8^+$ T cells didn't be affected after co-cultured with $CD11b^{hi}F4/80^{low}$ $TAMs^{CCL5+/+}$ and $CD11b^{hi}F4/80^{low}$ $TAMs^{CCL5-/-}$ cells. S00a8 and/or S100a9 (200ng/mL) were added in the contact co-cultured system for 24 hours, FACS quantification of apoptosis of $CD8^+$ T cells, for 7-AAD markers.

Data are represented as mean±SEM, results are representative of 3 independent experiments.

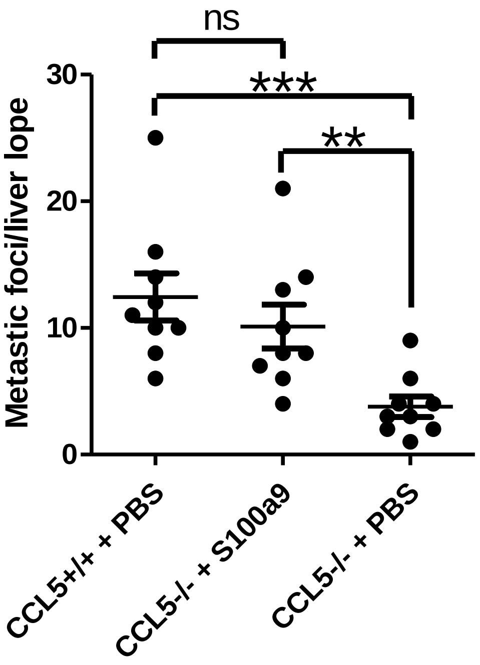

Supplementary figure 11. S100a9 promote the hepatic metastasis of colorectal cancer. Histologic identification of liver metastasis of $CT26^{+/+}$ and $CT26^{-/-}$ tumor cells in WT or KO mice and quantitation of liver metastasis determined by counting of foci per liver lobe. Each group contained three mice and each mouse counted three hepatic lobules.

ns, not statistically significant, ***P*<0.05, ****P*<0.001; Data are represented as mean±SEM.

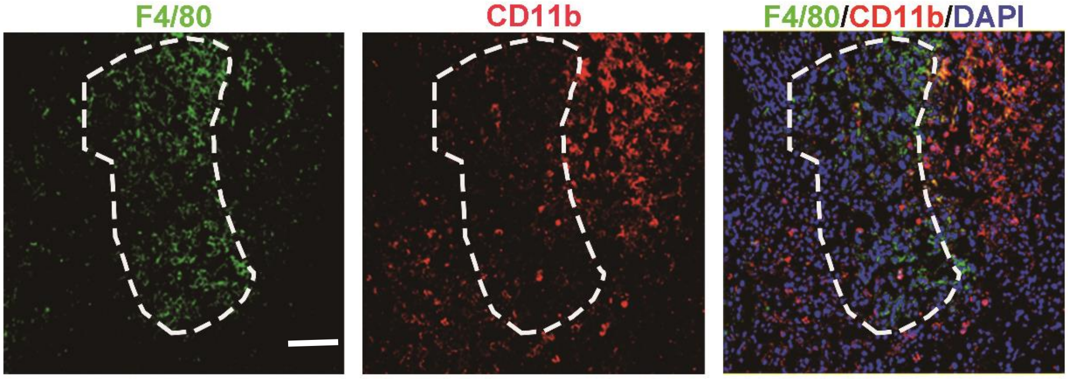

Supplementary figure 12. The CD11b^{hi} population is F4/80^{low}.

Tumor sections, separated from 3 weeks tumor bearing mice, were stained with F4/80, CD11b and 4`,6-diamidino-2-phenylindole(DAPI).

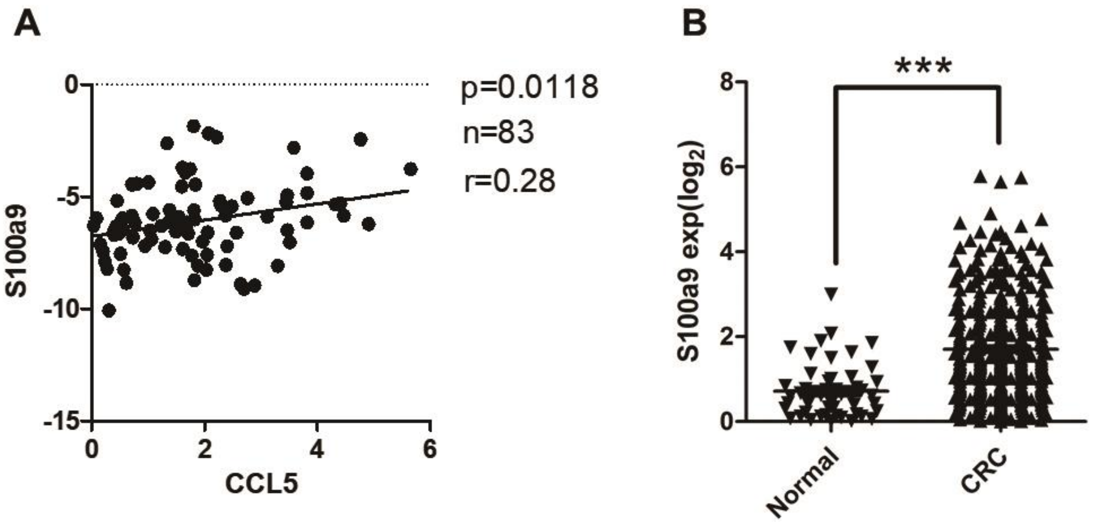

Supplementary figure 13. The expression level of CCL5 was positively correlated with S100a9 in CRC patients.

A. Correlation between CCL5 and S100a8/a9 mRNA expression in 83 CRC patients. B. the S100a9 gene expression in tumor part of 380 patients with CRC and 51 nontumor part of normal colon.

Data was generated from TCGA data base and analyzed using Pearson correlation analysis. ****P* <0.001, Data are represented as mean±SEM.

Supplemental Table

Table S1 Antibodies used in the paper

Name	Brand	Cat. #	Apps
Anti-Mouse CD28	R&D Systems	MAB4832-SP	Agonist activity
Anti-Mouse CD3	R&D Systems	MAB4841-SP	Agonist activity
Anti-Mouse CD4	BD Biosciences	561091	FCM
Anti-Mouse F4/80	eBioscience	11-4801-82	FCM
Anti-Mouse CD274(B7-H1)	eBioscience	85-46-5982-80	FCM
Anti-Mouse Ly6C PerCP	eBioscience	85-45-5932-82	FCM
Anti-Mouse CD62L FITC	eBioscience	85-11-0621-81	FCM
Anti-Human/Mouse CD44 APC	eBioscience	85-17-0441-81	FCM
Anti-Mouse CD69 PerCP	eBioscience	85-45-0691-80	FCM
Anti-Mouse CD8a APC	eBioscience	17-0081-82	FCM
Anti-Mouse CD223(LAG-3) FITC	eBioscience	85-11-2231-80	FCM
Anti-Mouse CD366(TIM3) PE	eBioscience	85-12-5871-81	FCM
Ati-mouse CD278(ICOS)PerCP	eBioscience	85-46-9940-80	FCM
Anti-Mouse CD11b APC	eBIoscience	85-17-0112-82	FCM
Mouse anti-CD279(PD-1) PerCP	eBioscience	46-9985-82	FCM
Mouse Anti-CD11b APC	eBioscience	17-0112-82	FCM
Mouse MHC Class II PE	eBioscience	12-5321-82	FCM
Mouse CD11c FITC	eBioscience	MA5-16877	FCM
Anti-Mouse Ly6G	eBioscience	12-5931-82	FCM
Mouse CD11b MAB	BD Biosciences	557394	IF
Hypoxyprobe™-1 Kits (MAb)	HPI	HPI-100Kit	IF
Mouse Anti-F4/80	abcam	ab186073	IF
Anti-mouse CD8a	R&D	MAB116	IF
Mouse Anti-CCL5	NOVUS	NBP1-19769	IHC
Human Anti-CCL5	abcam	ab9679	IHC
Human Anti-CD8a	abcam	ab199016	IHC
Anti-S100a8/a9 complex antibody	abcam	AB22506	IHC, IF
In vivo Mab anti-mouse PD-1(CD279) Ab	BioXcell	BE0146-5MG	i.p.
In vivo MAb anti-mouse CD8α	BioXcell	BE0061	i.p.
Mouse Anti-CCL5	Cell Signaling Technology	2989S	WB
Mouse Anti-GAPDH	Cell Signaling Technology	5174S	WB
Mouse β-Actin	Cell Signaling Technology	4970T	WB
